# Circulating microRNAs as potential diagnostic biomarkers for osteoporosis

**DOI:** 10.1038/s41598-018-26525-y

**Published:** 2018-05-30

**Authors:** Abdullah Y. Mandourah, Lakshminarayan Ranganath, Roger Barraclough, Sobhan Vinjamuri, Robert Van’T Hof, Sandra Hamill, Gabriela Czanner, Ayed A. Dera, Duolao Wang, Dong L. Barraclough

**Affiliations:** 1 Department of Musculoskeletal Biology, Institute of Ageing and Chronic Disease, University of Liverpool, The William Henry Duncan Building, 6 West Derby Street, Liverpool, L7 8TX United Kingdom; 2 Department of Clinical Biochemistry and Metabolic Medicine, The Royal Liverpool and Broadgreen University Hospital NHS Trust, Prescot Street, Liverpool, L7 8XP United Kingdom; 3 Department of Biochemistry, Institute of Integrative Biology, University of Liverpool, Biosciences Building, Crown Street, Liverpool, L69 7ZB United Kingdom; 4 Department Of Nuclear Medicine, The Royal Liverpool and Broadgreen University Hospital NHS Trust, Prescot Street, Liverpool, L7 8XP United Kingdom; 5 Department of Biostatistics and Eye and Vision Science, Faculty of Health and Life Sciences, The William Henry Duncan Building, 6 West Derby Street, Liverpool, L7 8TX United Kingdom; 6 Department of Clinical Sciences, Liverpool School of Tropical Medicine, Pembroke Place, Liverpool, L3 5QA United Kingdom; 7 Present Address: Department of Clinical Laboratory Sciences, College of Applied Medical Sciences, King Khalid University, Abha, Saudi Arabia; 8 Present Address: Al Hada Armed Forces Hospital, Taif, Saudi Arabia

## Abstract

Osteoporosis is the most common age-related bone disease worldwide and is usually clinically asymptomatic until the first fracture happens. MicroRNAs are critical molecular regulators in bone remodelling processes and are stabilised in the blood. The aim of this project was to identify circulatory microRNAs associated with osteoporosis using advanced PCR arrays initially and the identified differentially-expressed microRNAs were validated in clinical samples using RT-qPCR. A total of 161 participants were recruited and 139 participants were included in this study with local ethical approvals prior to recruitment. RNAs were extracted, purified, quantified and analysed from all serum and plasma samples. Differentially-expressed miRNAs were identified using miRNA PCR arrays initially and validated in 139 serum and 134 plasma clinical samples using RT-qPCR. Following validation of identified miRNAs in individual clinical samples using RT-qPCR, circulating miRNAs, hsa-miR-122-5p and hsa-miR-4516 were statistically significantly differentially-expressed between non-osteoporotic controls, osteopaenia and osteoporosis patients. Further analysis showed that the levels of these microRNAs were associated with fragility fracture and correlated with the low bone mineral density in osteoporosis patients. The results show that circulating hsa-miR-122-5p and hsa-miR-4516 could be potential diagnostic biomarkers for osteoporosis in the future.

## Introduction

Osteoporosis is the most common age-related bone disease worldwide, affecting more than 20 million individuals^1^. It is characterized by low bone mass and altered bone quality, which causes fragility and fractures and is usually clinically asymptomatic until the first fracture happens^2^. Osteoporosis often develops from osteopaenia, a condition of mild bone loss. The estimated annual cost of osteoporosis in the UK alone is over £1.7 billion^3^, and the costs are expected to increase on average by 25% by 2025 driven by aging populations^1^. At present, there is no national screening programme in the UK for bone diseases such as osteoporosis, thus, there is a pressing need for biomarkers to identify early osteoporosis.

MicroRNAs are critical molecular regulators in cells, which alter the expression of genes at a post-transcriptional level, by inhibiting the translation of particular mRNAs or inducing specific mRNA degradation^4^. Importantly, mature microRNAs can pass out of the cells and are found in blood, where they are stabilised either by being encapsulated in lipid vesicles or in a secreted complex with the protein argonaute^5^. Circulating microRNAs therefore show potential as valuable biomarkers for complex human conditions, such as cancer^6^ and diabetes^7^.

MicroRNAs regulate bone formation/resorption remodelling processes, bone cell growth, differentiation and function^8^. However, most microRNA studies have been carried out using cultured cells or animal model systems^9^. Changes in circulating microRNAs have been reported to be associated with osteoporotic fractures in a small number of studies. Seeliger *et al*.^10^ used miRNA microarrays to identify a panel of 9 up-regulated, but not down-regulated, serum miRNAs, which were associated with osteoporotic hip fractures in serum samples from 30 osteoporotic, compared to 30 non-osteoporotic patients. Garmilla-Ezquerra^11^ identified two miRNAs, from a panel of 760 miRNAs that were significantly differently expressed between fracture patients and hip osteoarthritis non-fracture controls in 38 patients. In a separate study, Panach *et al*.^12^ compared the levels of 179 miRNAs in sera from 15 bone fracture patients and 12 controls and identified three miRNAs that were upregulated sufficiently, relative to controls, to be suitable biomarkers in bone fracture. A further study screened 175 miRNAs in serum samples from 7 female patients with recent femoral neck fractures and 7 female controls. Six serum miRNAs exhibited significant variation with the presence of fracture and these were then tested on 11 control and 12 fracture patients, which confirmed the significant variation for three of the previously-selected miRNAs^13^. Kojican *et al*.^14^ analysed 187 miRNAs in 36 patients and 39 controls, divided into three groups, premenopausal females, post menopausal females and males. A panel of eight miRNAs was a good discriminator of fracture in all three groups. Yavropoulou *et al*.^15^ identified serum miRNAs, from a panel of 14 miRNAs selected from the literature, that were associated with low bone mass and vertebral fractures in 70 post menopausal women. In a wider-ranging study, combinations of 4 serum miRNAs were identified from a panel of 375 miRNAs, which could discriminate fracture status in type-two diabetics and in osteoporotic patients with high sensitivity and specificity using small groups of patients comprising 17–19 individuals^16^.

Other studies have compared differences in serum miRNAs between osteoporosis patients and various control groups. Li *et al*.^17^ studied three preselected miRNAs in plasma samples from 120 Chinese post-menopausal women in three groups of 40, normal, osteopaenia and osteoporosis. The levels of these three miRNAs, miR-21, miR-133a and miR-146 were significantly changed in the plasma of osteopaenia and osteoporosis patients compared to a normal group. Bedene *et al*.^18^ studied 9 miRNAs, which had shown promise in a previous screen based on bone and osteoarthritis expression, in 17 osteoporotic and 57 control subjects and identified miR-148a-3p as significantly higher in plasma of osteoporosis patients compared to controls. Plasma miR126-3p and miR423-5p correlated with measures of bone quality.

The aim of the present experiments was to investigate whether microRNAs in clinical samples of blood serum or plasma are associated with osteoporosis/low bone mineral density. Circulatory microRNAs associated with osteoporosis were identified initially on a non-selective basis using advanced PCR arrays containing 370 serum miRNAs and the identified differentially-expressed microRNAs were validated in over 139 clinical specimens using RT-qPCR. The resulting miRNAs could lead to a novel diagnostic tool for osteoporosis in the future.

## Results

### Characteristics of clinical samples and RNA quality check

A total of 161 participants were recruited (Table 1). However, 22 participants, who were under the age of 40 years, were not included in the data analysis, including 18 of the healthy controls, 2 of the osteopaenia and 2 of the osteoporosis patients. The remaining 139 participants who were over 40 years old were included in the analysis. These consisted of 12 normal controls, 76 osteopaenia and 51 osteoporosis patients. Based on the Bone Mineral Density (BMD) and the *T*-Score, participants were classified into 5 sub-groups: A. 12 (11 female/1 male) Non-osteoporosis controls (BMD *T*-Score >−1). B. 61 (52 female/9 male) Osteopaenia without fracture (BMD *T*-Score −2.4 to −1). C. 15 (13 female/2 male) Osteopaenia with fracture (BMD *T*-Score −2.4 to −1), D. 33 (27 female/6 male) Osteoporosis without fracture (BMD *T*-Score ≤−2.5) and E. 18 (16 female/2 male) Osteoporosis with fracture (BMD *T*-Score ≤−2.5). The average age of healthy control participants was 67 years ± SD 9.6. The average age of osteopaenia patients without fracture was 65.6 years ± SD 9.5, osteopaenia patients with fracture was 67 years ± SD 9.5, osteoporosis patients without fracture was 68.6 years ± SD 10 and osteoporosis patients with fracture was 70 years ± SD 10, respectively. Among those patients with low bone mass (BMD *T*-score <−1), 94 patients including 79 post-menopausal women and 15 men were without fractures and 33 patients including 29 post-menopausal women and 4 men were with fractures. These 33 subjects suffered 65 fractures, which were located as follows: ankle/foot, 4; wrist, 14; vertebrae, 23; ribs, 12; pelvis, 1; hip, 5; shoulder, 4. 81% of participants were females. 10 out of 11 female controls, 60 out of 65 female osteopaenia and 41 out of 43 female osteoporosis patients were post-menopausal.

**Table 1:** Summary of Characteristics of Clinical Samples.

Clinical Category	Non-Osteoporosis Control (NOPC)	Osteopaenia*		Osteoporosis*	
		Osteopaenia without fracture	Osteopaenia with fracture**	Osteoporosis without fracture	Osteoporosis with fracture**
Total number of participants recruited (Female/Male)	30 (20/10)	63 (53/10)	15 (13/2)	34 (28/6)	19 (17/2)
Total nmber of >40 years old participants (Female /Male) included in the analysis	12 (11/1)	61 (52/9)	15 (13/2)	33 (27/6)	18 (16/2)
Mean age years (Mean ± SD)	67 ± 9.6	65.6 ± 9.5	67 ± 9.5	68.6 ± 10	70 ± 10
BMD (g/cm^2^) Mean ± SD	0.96 ± 0.07	0.83 ± 0.10	0.88 ± 0.12	0.7 ± 0.07	0.7 ± 0.1
*T*-Score Lumbar Spine (L2-L4) Mean ± SD	0.6 ± 1.4	−1.2 ± 0.9	−1.1 ± 1.1	−2.7 ± 0.95	−2.78 ± 1

^*^Four osteopaenia patients and 1 osteoporosis patient were reported as suffering from coeliac disease, 3 osteopaenia and 1 osteoporosis patients were reported to suffer from asthma and were on regular steroid medication. One osteopaenia patient was suffering from lung cancer, 1 osteopaenia patient and 4 osteoporosis patients had type-2 diabetes and 1 osteopaenia patient was suffering from type-3c (pancreatogenic) diabetes. 5 osteopaenia and 3 osteoporosis patients suffered from hypertension.

^**^Fractures occurred between 1 month and 2 years before the collection of blood samples.

17 osteopenia and 27 osteoporosis patients were receiving Bisphophonates, including Alendronate, Ibandronate, Risedronate and Zoledronic acid, 2 osteoporosis patients received Denosumab.

RNAs were isolated from 161 serum and 142 plasma samples with average RNA yield of 1437.1 ± 680.8 ng and 1297 ± 607 ng, respectively. The purified RNAs showed distinctive small RNA bands using an Agilent 2100 Bioanalyzer. RNAs that did not pass quality check were not included in this study. Therefore, only high quality RNAs purified from 139 serum samples and 134 plasma samples were used for RT-qPCR analysis.

### Differentially-expressed circulating MicroRNAs were identified using miRNA PCR arrays

**Figure 1:**
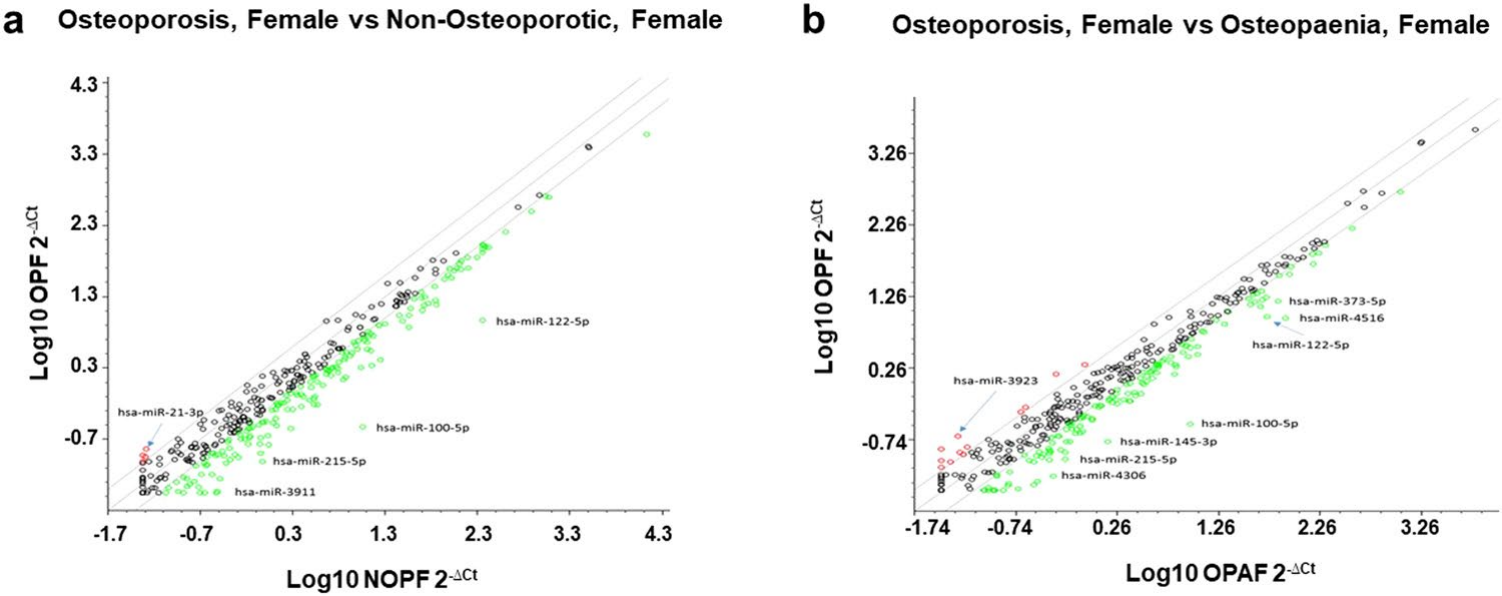
Analysis of differentially-expressed microRNAs between osteoporosis, osteopaenia and non-osteoporosis female groups. Scatter plots show the fold change (log10 (2^−ΔCt^) of the level of 370 microRNAs. Panel (a) between osteoporotic and non-osteoporotic females. Panel (b) between osteoporotic and osteopaenia females. MicroRNAs over-expressed by 2-fold are shown in red and those under-expressed by 2-fold are shown in green.

Initially, circulating microRNAs from pooled serum samples were profiled using the Human Serum and Plasma miRNA PCR arrays containing 370 mature miRNAs. Differentially-expressed microRNAs were identified between osteoporosis, osteopaenia and non-osteoporosis groups using the log10 (2^−ΔCt^) fold change of the levels of microRNAs between the osteoporosis group and the non-osteoporotic group and between the osteoporosis group and the osteopaenia group (Fig. 1).

Fifteen differentially-expressed microRNAs (over 2-fold changes) were identified between the osteoporosis, female group and the non-osteoporotic, female group and 25 up or down (over 2-fold changes) differentially-expressed microRNAs were identified between the osteoporosis, female group and the osteopaenia, female group (Table 2).

**Table 2:** Differentially-expressed MicroRNAs in the female osteoporosis group compared with non-osteoporosis group and osteopaenia group using microRNA PCR array.

MicroRNA	Fold Change Osteoporosis/Non-osteoporosis	MicroRNA	Fold Change Osteoporosis/Osteopaenia
hsa-miR-21-3p	2.8	hsa-miR-3923	4.1
hsa-miR-1231	2.3	hsa-miR-4258	4.0
hsa-miR-100-5p	−38.3	SNORD61	3.3
hsa-miR-122-5p	−24.1	hsa-miR-196b-3p	2.8
hsa-miR-215-5p	−9.8	hsa-miR-485-5p	2.8
hsa-miR-3911	−8.3	hsa-miR-1193	2.4
hsa-miR-1290	−6.9	hsa-miR-2467-3p	2.4
hsa-miR-194-5p	−6.8	hsa-miR-1281	2.3
hsa-miR-145-3p	−6.5	hsa-miR-4274	2.3
hsa-let-7a-3p	−6.1	hsa-miR-100-5p	−32.1
hsa-miR-4306	−5.8	hsa-miR-4516	−9.1
hsa-miR-10b-5p	−5.5	hsa-miR-145-3p	−8.8
hsa-miR-365b-3p	−5.4	hsa-miR-4306	−7.5
hsa-miR-200b-3p	−5.1	hsa-miR-548e-3p	−6.4
hsa-miR-99a-5p	−5.0	hsa-miR-206	−6.3
		hsa-miR-215-5p	−5.8
		hsa-miR-122-5p	−5.7
		hsa-miR-3911	−5.2
		hsa-miR-548d-5p	−4.7
		hsa-miR-373-5p	−4.5
		hsa-miR-99a-5p	−4.4
		hsa-miR-375	−3.9
		hsa-miR-450a-5p	−4.2
		hsa-miR-143-3p	−4.0
		hsa-miR-1290	−3.6

### Differentially-expressed microRNAs associated with osteoporosis patients

**Figure 2:**
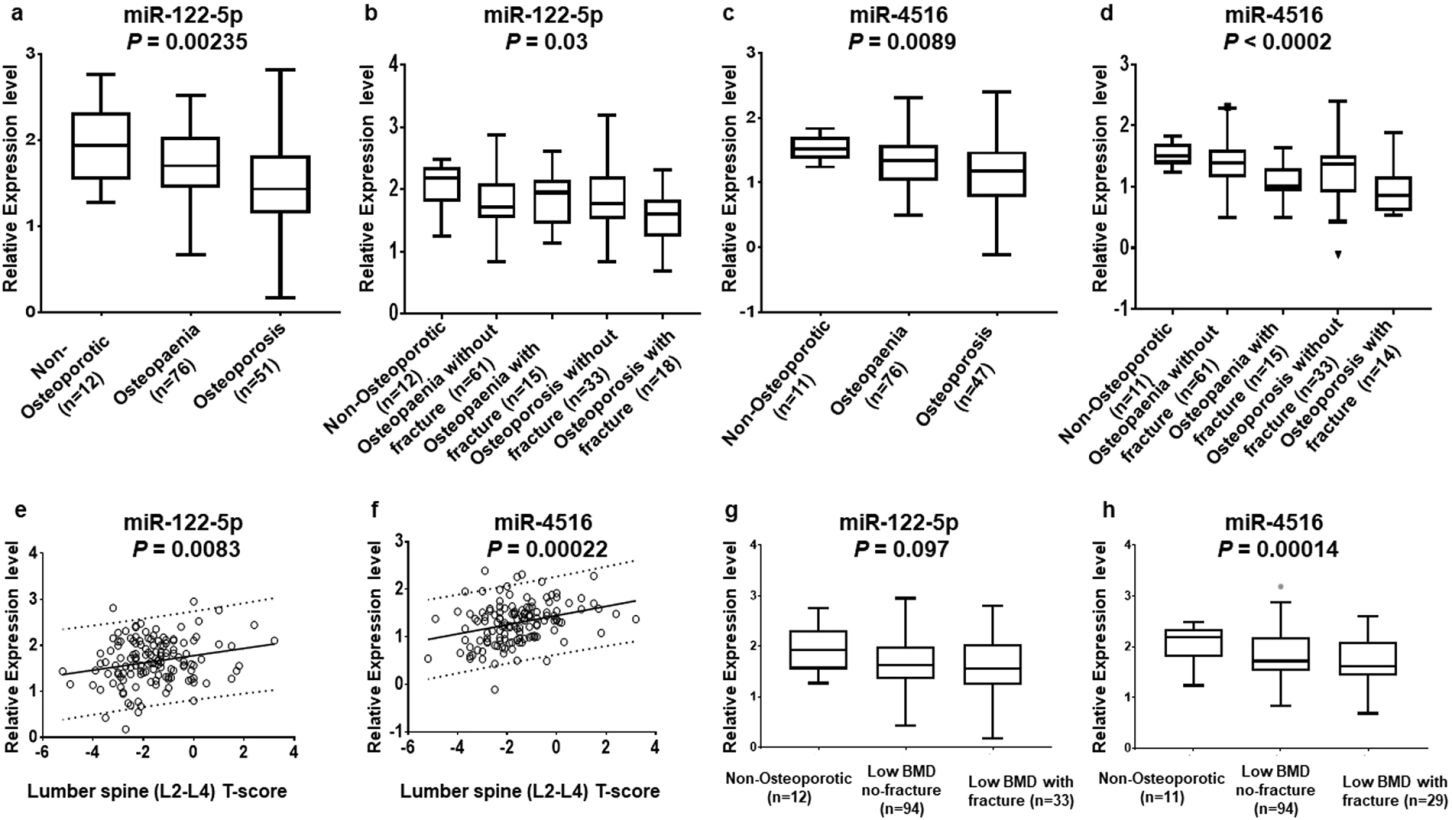
Identification of microRNAs associated with osteoporosis. Box plots show the levels of microRNAs determined by RT-qPCR in clinical samples associated with the development of osteoporosis. The data includes 20 male participants, comprising one male control, 11 with osteopaenia and 8 with osteoporosis. These numbers were too small to warrant separate analysis. Panel (a), box plots show the levels of hsa-miR-122-5p among non-osteoporotic controls, osteopaenia and osteoporosis patients (*P* = 0.00235); Panel (b), box plots show a statistically significant correlation of hsa-miR-122-5p within the osteoporosis patients who had fracture (*P* = 0.03); Panel (c), box plots show the levels of hsa-miR-4516 among non-osteoporotic controls, osteopaenia and osteoporosis patients (*P* = 0.0089); Panel (d), box plots show that hsa-miR-4516 is associated with osteoporosis patient with fracture (*P* < 0.0002); Panel (e), the levels of hsa-miR-122-5p in clinical samples significantly increased with increasing lumbar spine (L2-L4) of the subject (*P* = 0.0083); Panel (f), the levels of hsa-miR-4516 in clinical samples significantly increased with increasing lumbar spine (L2-L4) of the subject (*P* = 0.00022); Panel (g), box plots show the levels of hsa-miR-122-5p among non-osteoporotic controls, low BMD patients without fracture and low BMD patients with fracture (*P* = 0.097); Panel (h), box plots show the levels of hsa-miR-4516 among non-osteoporotic controls, low BMD patients without fracture and low BMD patients with fracture (*P* = 0.00014).

The levels of microRNAs identified as being up or down-regulated between groups by miRNA PCR arrays (Table 2) were validated on 139 serum samples and 134 plasma samples using RT-qPCR. The results demonstrated that hsa-miR122-5p and hsa-miR4516 are present at significantly different levels between non-osteoporotic control, osteopaenia and osteoporosis patients as shown in Fig. 2. The levels of miR122-5p in serum are much lower in osteoporosis patients compared with non-osteoporotic control and osteopaenia patients (*P* = 0.00235) (Fig. 2a), particularly in osteoporosis patients with fracture (*P* = 0.03) (Fig. 2b). The levels of miR4516 in osteoporosis plasma samples decreased compared to non-osteoporotic control and osteopaenia patients (*P* = 0.0089) (Fig. 2c). The levels of miR-4516 also tended to be lower among osteoporosis patients with fracture (*P* < 0.0002) (Fig. 2d). The results suggest that the decreasing levels of hsa-miR122-5p and hsa-miR-4516 in clinical samples might be associated with the development of osteoporosis in these patients. Other differentially-expressed miRNAs, such as miR100-5p, did not show significant variation by the ANOVA statistical test between the 5 groups, non-osteoporosis, osteopaenia without fracture, osteopaenia with fracture, osteoporosis without fracture and osteoporosis with fracture and were not studied further.

Linear regression analysis showed that there was no significant association between age and the level of circulating microRNAs, miR-122-5p, Pearson r = 0.013, 95%CI (−0.14 to 0.17), *P* (two-tailed) = 0.874; and miR-4516, Pearson r = 0.069, 95%CI (−0.23 to 0.097), *P* (two-tailed) = 0.413.

It is possible that the changes in circulating miRNAs, hsa-miR-122-5p and hsa-miR-4516 reported here, arose from subjects suffering from other diseases. However, using ANOVA statistical data analysis, the results showed that exclusion of patients with other diseases from the analysis did not change the significance of the results (miR-122-5p, when patients with other diseases were excluded, *P* = 0.0067, when patients with other diseases were included, *P* = 0.0024; miR4516, excluded other diseases, *P* = 0.006, other diseases included, *P* = 0.0089). Further, pairwise comparisons of three groups: normal control, osteopaenia and osteoporosis, using Bonferroni correction, the results showed that the relationships among the three groups were not affected by the inclusion of patients with other diseases (Supplemental Table 1).

To find out whether the patients on anti-osteoporosis therapy were affecting the overall results shown in Fig. 2, the effect of removing patients on anti-osteoporosis treatment from the data analysis was determined. Again, using ANOVA analysis of the three groups shown in Fig. 2, the results showed that their removal had a slight effect on the results, but did not affect the overall significance of the results (miR-122-5p, patients on anti-osteoporotic treatment included, *P* = 0.0024, patients on anti-osteoporotic treatment excluded, *P* = 0.0154; miR-4516, patients on anti-osteoporotic treatment included, *P* = 0.0089; patients on anti-osteoporotic treatment excluded, *P* = 0.014). Further, pairwise comparisons of the three groups, normal control, osteopaenia and osteoporosis, using Bonferroni correction, showed that the relationships between osteopaenia or osteoporosis groups and the control group were not affected by removing patients with anti-osteoporotic treatment (Supplemental Table 1). These results clearly show that the inclusion of patients with anti-osteoporotic treatment did not affect the original results.

To assess the effect of the male participants on the overall results shown in Fig. 2, the male participants were removed from the data analysis. Using ANOVA analysis of the three groups shown in Fig. 2, the results showed that their removal did not affect the overall significance of the results (miR-122-5p males included, *P* = 0.0024, males excluded, *P* = 0.0021; miR-4516, males included, *P* = 0.0089; males excluded, *P* = 0.0026). The pairwise comparisons of the osteopaenia and osteoporosis groups with the controls using the Bonferroni correction were not affected by removing the male participants (Supplementary Table 1). Thus, the inclusion of the males did not affect the reported results for hsa-miR-122-5p and hsa-miR-4516.

The levels of hsa-miR122-5p and hsa-miR4516 in clinical samples also independently showed a strong significant correlation with BMD lumbar spine *T*-score (r = 0.22, *P* = 0.0083 and r = 0.31, *P* = 0.00022, respectively, Fig. 2e,f). Whereas miR-122-5p did not distinguish between low BMD patients with and without fracture (Fig. 2g), the levels of miR-4516 in the low BMD (osteopaenia and osteoporosis) with fracture patients were highly significantly lower (*P* = 0.00014) than low BMD without fracture patients and non-osteoporotic controls (Fig. 2h). The results indicate that the levels of miR4516 in clinical samples are strongly linked with those from osteopaenia and osteoporosis patients with low BMD, who have experienced fracture.

In the present experiments, hsa-miR-122-5p and hsa-miR-4516 have been identified in the serum/plasma from osteoporosis patients. The precise normal cellular locations of these miRNAs in bone cells associated with osteoporosis is presently unknown. OMIM disease associations using the Enrichr database^19^ (http://amp.pharm.mssm.edu/Enrichr) showed that both miRNAs, hsa-miR-122-5p and hsa-miR-4516, were associated with osteoporosis, amongst other disorders. Potential target mRNAs for hsa-miR-122-5p and hsa-miR-4516 have been identified bioinformatically using four different algorithms within the miRWalk2.0 database, namely miRWalk, miRanda, RNA22 and Targetscan. Targetscan predicts biological targets of miRNAs by searching for the presence of conserved 8mer, 7mer and 6mer sites that match the seed region of each miRNA^20^. Predictions are ranked based on the predicted efficacy of targeting as calculated using a cumulative weighted score based on 14 features of the sites as described by Agarwal *et al*.^21^. A total of 10,891 and 7,641 putative mRNA targets were identified for hsa-miR-122-5p and hsa-miR-4516, respectively. In order to identify which of these potential targets might be relevant to osteoporosis, the miRWalk2.0 database of mRNAs was interrogated with the search term osteoporosis [DOID: 11476] and 89 mRNAs encoding osteoporosis-associated proteins were identified. These 89 mRNAs were then checked for hsa-miR-122-5p and hsa-miR-4516 target sequences within their 3′-untranslated regions using the 4 algorithms within miRWalk2.0 and possible roles in osteoblast/osteoclast metabolism in the context of osteoporosis identified. Any mRNAs containing interaction sites for hsa-miR-122-5p and hsa-miR-4516 sequences, predicted by at least 2 out of the 4 algorithms, were recorded. Twenty and 21 of the 89 putative osteoporosis-related mRNAs were identified as targets for hsa-miR-122-5p and hsa-miR-4516, respectively (Supplemental Table 2). Eight of the osteoporosis-related mRNAs were targets of both hsa-miR-122-5p and hsa-miR-4516, namely, BMP2 inducible kinase (BMP2K), follicle stimulating hormone beta subunit (FSHB), insulin-like growth factor 1 receptor (IGF1R), parathyroid hormone-like hormone (PTHLH), runt-related transcription factor 2 (RUNX2), Secreted protein acidic and cysteine rich (SPARC), TSC22 domain family member 3 (TSC22D3) and vitamin D (1,25-dihydroxy vitamin D3) receptor (VDR). Other common potential osteoporosis-related targets of hsa-miR-122-5p and hsa-miR-4516 were members of the same protein family, namely cytochrome P450 family 3 subfamily A member 4 (CYP3A4), mitogen activated protein kinase 1 (MAPK1) and cannabinoid receptor 2 (CNR2) for hsa-miR122-5p and cytochrome P450 family 17 subfamily A member 1 (CYP17A1), cytochrome P450 family 19 subfamily A member 1 (CYP19A1), mitogen activated protein kinase 3 (MAPK3) and cannabinoid receptor 1 (CNR1) for hsa-miR-4516, showing that miRNAs hsa-miR-122-5p and hsa-miR-4516 potentially regulate osteoporosis-related genes with a number of common target mRNAs and pathways associated with bone metabolism.

### Diagnostic value of microRNAs for osteoporosis

**Figure 3:**
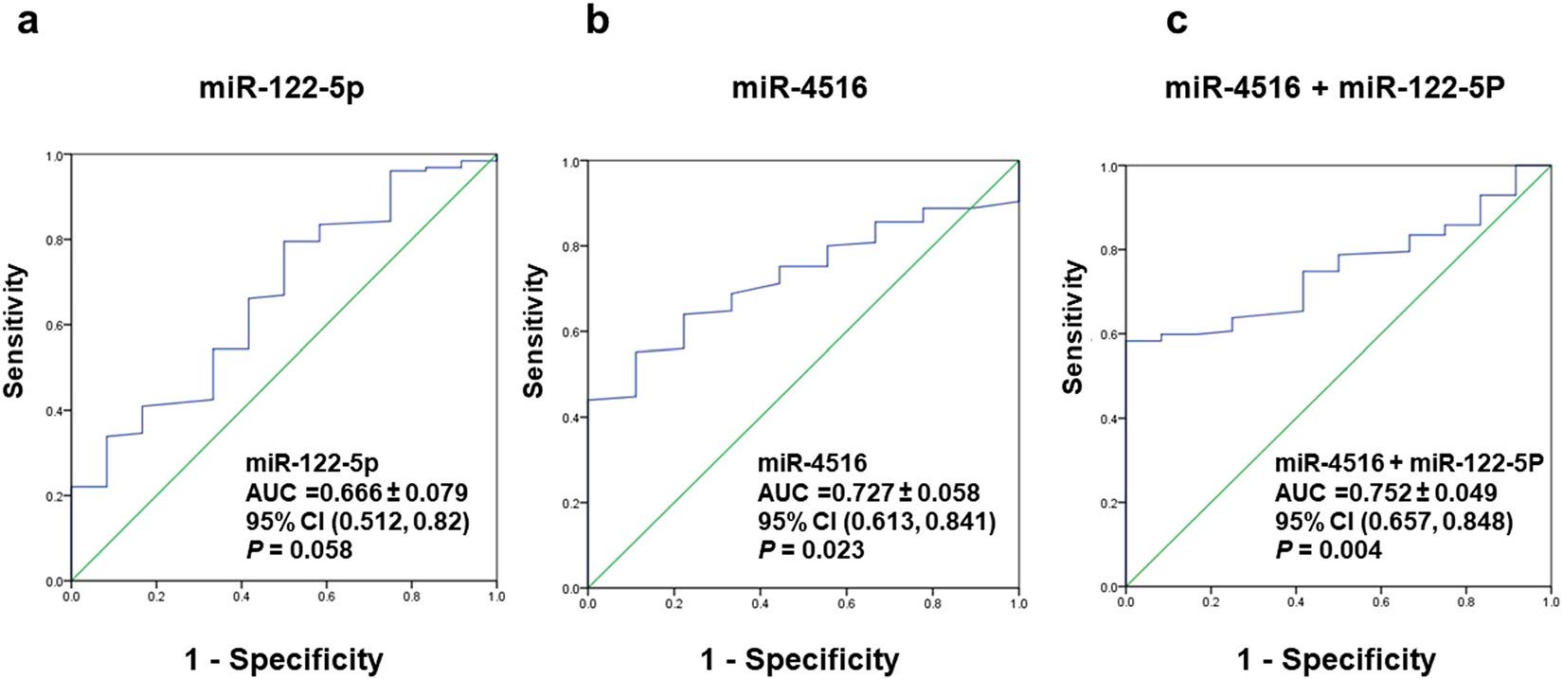
Diagnostic value of hsa-mi-R122-5p and hsa-miR-4516 for osteoporosis. ROC curves show that AUC of hsa-miR-122-5p in clinical samples for osteoporosis (AUC = 0.666, 95% CI = 0.512 to 0.82, *P* = 0.058) (Panel a), an acceptable AUC discrimination of hsa-miR-4516 in clinical samples for osteoporosis (AUC = 0.727, 95% CI = 0.613 to 0.841, *P* = 0.023) (Panel b), and a good AUC of a combination of miR-122-5p and miR-4516 in clinical samples for osteoporosis (AUC = 0.752, 95% CI = 0.657 to 0.848, *P* = 0.004) (Panel c). AUC = Area under the curve. CI = Confidence interval.

Diagnostic values for miR-122 in clinical samples for osteoporosis patients were not found to be significant in this data (AUC = 0.666, *P* = 0.058) (Fig. 3a). There were acceptable diagnostic values for the levels of hsa-miR-4516 in clinical samples for osteoporosis patients (AUC = 0.727,*P* = 0.023) (Fig. 3b). The sensitivity and specificity for miR-4516 in clinical samples were 71% and 62%, respectively. However, when both microRNAs were combined together, there was a much stronger diagnostic value for osteoporosis (AUC = 0.75, *P* = 0.004) than either separately (Fig. 3c). The results suggest that both microRNAs, miR-122-5p and miR-4516 could be used together to increase diagnostic value for osteoporosis.

## Discussion

MiRNA PCR arrays have identified 15 up- and down-regulated miRNAs from pooled serum samples between the osteoporosis group and the non-osteoporotic control group, and 25 miRNAs between the osteoporosis group and the osteopaenia group. After RT-qPCR validation in patients/normal controls, two miRNAs, hsa-miR-122-5p and hsa-miR-4516 were significantly down-regulated in clinical blood samples from osteoporosis patients vs non osteoporosis subjects or between osteoporosis patients and osteopaenia patients. Noticeably, hsa-miR-122-5p was detectable in serum samples, but hsa-miR-4516 was detectable in plasma samples, suggesting that the clinical specimen sample type seems to influence the detection of the miRNAs. In contrast to the present study, hsa-miR-122-5p has been reported recently to be up-regulated in the serum of patients suffering hip fracture by Panach *et al*.^12^. Panach *et al*. reported that hsa-miR-122-5p was upregulated in 15 female patients suffering from femoral neck fracture compared to 12 patients suffering from osteoarthritis. However, our study compared 51 osteoporosis and 76 osteopaenia patients, compared to 12 normal controls. These differences between the nature of the control subjects and differences in the sample sizes could account for the different result. Increased level of circulating miR-122-5p, in combination with other miRNAs, has been reported to be of diagnostic value in knee osteoarthritis^22^ and miR-122-5p is one of a group of miRNAs that were downregulated when human adipose-derived mesenchymal stem cells were stimulated to differentiate with vitamin D3^23^. There have been no previous reports of circulating miR-4516 being associated with bone metabolism or osteoporosis. In a separate study, a panel of up-regulated, but not down-regulated, miRNAs was identified to be associated with patients with osteoporotic fractures compared with a control group of patients with non-osteoporotic fractures from only a small group of 20 serum samples and a screen of only 83 microRNAs^10^.

A number of other studies have identified serum/plasma miRNAs that are associated with osteoporosis/low BMD/fractures, however, none of these identified hsa-miR-122-5p or hsa-miR-4516. Kojican *et al*. analysed 187 miRNAs in 36 patients and 39 controls^14^, Bedene *et al*. studied 9 miRNAs, which had shown promise in a previous screen, in 74 postmenopausal women, of which 17 were osteoporotic and 57 were controls^18^, Li *et al*. studied only three preselected miRNAs in 120 Chinese post-menopausal women in three groups of 40, normal, osteopaenia and osteoporosis^17^, Yavropoulou *et al*.^15^ identified serum miRNAs that were associated with low bone mass and vertebral fractures in 70 post menopausal women, from a panel of just 14 miRNAs selected from the literature. However, all these studies used non-random selection of miRNAs, in contrast to the present study, in which a larger number of 370 microRNAs were screened randomly using miRNA PCR arrays. miRNA arrays were used in separate studies to identify differentially-expressed miRNAs associated with osteoporotic fractures in 20 patients^10^ or differentially-expressed between fracture patients and hip osteoarthritis non-fracture controls in 38 patients^11^, or tested on a total of 19 female patients with recent femoral neck fractures and 18 female controls^13^, in contrast to the larger number of 139 participants in the present study.

Anastasilakis *et al*.^24^ reported that the levels of serum miRNAs were altered by anti-osteoporotic treatment (such as Teriparatide or Denosumab) in postmenopausal female patients. Although none of the patients in the present study were treated with Teriparatide, two subjects were treated with Denosumab. However, when patients receiving anti-osteoporotic treatment (Denosumab and Bisphosphonates) were excluded from the analyses, there was no effect on the overall significance of the results.

Possible tissue sources of miR-122-5p and miR-4516 were examined. Both hsa-miR-122-5p and hsa-miR-4516 were identified in osteosarcoma cells, which have been used as a model for osteoblast cells^25^. For miR-122-5p, The Human miRNA Tissue Atlas^26^ (website https://ccb-web.cs.uni-saarland.de/tissueatlas/) Variance Stabilised Normalisation (VSN) relative data^27^ showed that miR-122-5p is expressed in liver and bone, as well as a number of other tissues, but hardly present in bone marrow. However, recently, it has been reported in rats that microvesicles produced by resting zone chondrocytes are over 800-fold enriched in hsa-miR-122-5p than the producing cells^28^, whereas miR-122-5p is not present in microvesicles from growth zone chondrocytes. This observation raises the novel possibility that the changes in serum hsa-miR-122-5p levels in osteoporosis/osteopaenia patients might reflect changes in chondrocyte-derived microvesicles in the blood.

miR-4516 is broadly associated amongst tissues (Tissue Atlas;^26^), but is found particularly in the oesophagus and in keratinocytes, where it has been associated with cell apoptosis^29^. The present experiments are the first to identify miR-4516 in osteosarcoma cells and the first to link reduced levels of plasma miR-4516 with osteoporosis/osteopaenia in patients.

Whilst miR-122-5p has been previously located to bone tissue (Tissue Atlas), there is no information on the cellular location of hsa-miR-122-5p or hsa-miR-4516 within the various cell types of bone tissue. In the present work, bioinformatic analysis showed that both hsa-miR-122-5p and hsa-miR-4516 targeted complementary sequences in the 3′ non-coding regions of 20 and 21 mRNAs, respectively, which encode proteins that had been associated previously with osteoporosis. Eight of these mRNAs were common to both hsa-miR-122-5p and hsa-miR-4516. Of these 8 common genes, 7 have been shown previously to occur in osteoblasts: bone morphogenetic protein inducible kinase (BMP2K^30^), follicle stimulating hormone beta subunit (FSHB^31^), insulin-like growth factor 1 receptor (IGF1R^32^), runt-related transcription factor 2 (RUNX2^33^), secreted protein acidic and cysteine rich (SPARC^34^), TSC22D3 domain family member 3 (TSC22D3^35^) and vitamin D receptor (VDR^36^). Of the remaining 12 mRNA targets of hsa-miR-122-5p, 6 encoded proteins were previously-described as bone markers or to occur in osteoblasts or osteoclasts (cannabinoid receptor 2 (CNR2^37^), alkaline phosphatase, liver/bone/kidney (ALPL^38^), inorganic pyrophosphate transport regulator (ANKH^39^), CD44^40^, estrogen receptor 1 (ESR1^41^) and low density lipoprotein receptor related protein 6 (LRP6^42^). Of the remaining 13 mRNA targets of hsa-miR-4516, cannabinoid receptor 1 (CNR1^43^) and androgen receptor (AR^44^) have been specifically associated with human osteoblasts/osteoclasts. These results associate microRNAs hsa-miR-122-5p and hsa-miR-4516 with mRNAs expressed in osteoblast or osteoclast cells and raise the possibility that these two miRNAs may also localise to these cell types in humans.

In the present experiments, down-regulation of these two miRNAs, hsa-miR-4516 and hsa-miR-122-5p, in the blood samples was also significantly associated with the occurrence of osteoporotic fractures and reduced bone mineral density *T*-score. ROC analysis for hsa-miR-4516 showed an AUC of 0.727 (95% CI 0.613 to 0.841, *P* = 0.023) compared with 0.666 (95% CI 0.512 to 0.82, *P* = 0.058) for hsa-miR-122-5p, however, the AUC was improved when both microRNAs were included (AUC = 0.752, 95% CI 0.657 to 0.848, *P* = 0.004), suggesting that these microRNAs might have utility for further development as biomarkers for osteoporosis.

## Materials and Methods

### Clinical samples

The study conformed to the principles of the Helsinki Declaration. Ethical approval was obtained from the England Health Research Authority National Research Ethics Service Committees North West-Greater Manchester West [REC reference 11/NW/0593] and East of England-Essex [REC reference 15/EE/0051] prior to commencement of the study. The inclusion criteria for this study were patients who were over 18 years old and able to consent on their own, and either suffering osteopaenia or osteoporosis or healthy volunteers as a control group. Exclusion criteria for this study were all participants who were under 18 years old or unable to consent on their own or suffering from a disease unrelated to osteoporosis. However, patients with diseases not unrelated to osteoporosis were included. Informed consent was obtained from all participants prior to sample collection. Clinical samples were obtained from participants, including local health volunteers and patients who were referred to the Royal Liverpool Broadgreen University Hospital NHS Trust based in Liverpool, either presenting at the bone clinic or at the Department of Radiology for a bone mineral density (BMD) scan. Following informed consent, blood samples were obtained by a qualified phlebotomist at the Royal Liverpool Broadgreen University Hospital NHS Trust. All samples were given a unique barcode and were processed immediately in the laboratory. Serum or plasma samples were prepared by centrifugation at 2,500 × g for 30 min at room temperature initially and collected supernatants were clarified by being centrifuged at 14,000 × g for 30 min at 4 °C. Aliquots of supernatants were stored frozen at −80 °C for later RNAs extraction.

### Isolation and purification of microRNAs from serum and plasma samples

Micro RNAs were isolated and purified from serum or plasma using a combination of TRIzol® LS reagent and QIAGEN miRNeasy Mini Kit according to the manufacture’s recommendations. Briefly, a 400 µL aliquot of a serum or a plasma sample was mixed with 1.2 mL TRIzol® LS reagent with rigorous vortexing and incubated at room temperature for 15–30 min. Glycogen (10 mg/mL) was added to a final concentration of 15 ng/µL to improve the yield of RNA and synthetic miRNA-39 from *Caenorhabditis elegans* (Sysn-cel-miR-39) (QIAGEN) was added to a final concentration of 15 pmol/L as a spike-in control. After adding 320 µL chloroform (99.5%), the mixture was vortexed vigorously for 30 s, incubated on ice for 10 min and centrifuged for 30 min at 14,000 × g at 4 °C. The clear upper aqueous solution containing RNA was precipitated by adding 1.5 volume of 100% ethanol. Purification of extracted total RNA was performed using miRNeasy columns (QIAGEN) according to the manufacturer’s recommendation. Finally, the resulting purified RNAs were eluted in 30 µL of RNase-free water and stored frozen at −80 °C until used. RNA concentrations and purity were measured initially using a Thermo Scientific NanoDrop™ 2000 spectrophotometer and validated using an Agilent 2100 analyser.

### microRNAs PCR array analysis

microRNA PCR array analysis was performed using pooled serum groups from human subjects, including a pool of 4 non-osteoporotic female samples (NOPFP1), a pool of 8 osteopaenia female samples (OPAFP3) and a pool of 9 osteoporosis female samples (OPFP4). The expression of MicroRNAs among these pools was profiled using the Human Serum & Plasma miRNA PCR Array MIHS‐3106Z (QIAGEN), which represented 370 mature miRNAs and six reference genes, SNORD61, SNORD68, SNORD72, SNORD95, SNORD96A and RNU6-6P and two RNA and PCR quality controls, including reverse transcription control and positive PCR control. The Real-Time miRNA PCR array contains a specific set of primers that recognize human serum/plasma microRNAs. The purified RNA samples were converted to cDNA, then added to the Real-Time miRNA PCR arrays and the primer set allowed amplification and identification of microRNAs present in the biological samples. The levels of microRNAs from the array were calculated from the Ct value after normalization with two control microRNAs, SNORD96A and RNU6-6P, using the 2^−ΔΔCT^ method^45^. All Ct values greater than 30 obtained from the microRNA PCR array were considered to be below the detection level of the reaction.

### Quantitation of microRNA levels using real-time quantitative PCR [RT-qPCR]

Real-time quantitative PCR (RT-qPCR) of circulating microRNAs identified from the PCR arrays was carried out on 139 serum and 134 plasma samples. Briefly, 100 ng of purified RNA was reverse transcribed in 20 µL reactions using the miScript reverse transcription kit (Qiagen) according to manufacturer’s recommendation. Reverse transcriptase was replaced by RNase-free water to provide a no-DNA control for later RT-qPCR. The resulting reverse transcription products were diluted at a ratio at 1:3 prior to RT-qPCR amplification. All RT-qPCR analyses were conducted in duplicate in 10 µL volume using the miScript SYBR Green PCR kit (QIAGEN) with a Roche LightCycler 96 Real-Time PCR system (Roche). A no-reverse-transcriptase control sample was included for each RT-qPCR amplification. All primers used for RT-qPCR in this study are listed in Supplemental Table S3. The relative levels of each microRNA were determined from the Ct value after normalization with two control molecules, SNORD96A and RNU6–6P, using the 2^−ΔΔCT^ method^45^. All Ct values obtained from RT-qPCR greater than 35 were considered to be below the detection level of the reaction.

### Bioinformatic analysis of intracellular target mRNAs of miRNAs

OMIM disease associations of selected miRNAs were identified using the Enrichr database^19^ (http://amp.pharm.mssm.edu/Enrichr). Potential target mRNAs for microRNAs were identified bioinformatically using four different algorithms within the miRWalk2.0 database (http://zmf.umm.uni-heidelberg.de/mirwalk2/)^46^, namely miRWalk, miRanda, RNA22 and Targetscan. Targetscan predicts biological targets of miRNAs by searching for the presence of conserved 8mer, 7mer and 6mer sites that match the seed region of each miRNA^20^. Predictions are ranked based on the predicted efficacy of targeting, as calculated using a cumulative weighted score based on 14 features of the sites as described by Agarwal *et al*.^21^. Potential target mRNAs were selected if they were predicted by 2 of the 4 algorithms.

### Statistical analysis

After logarithmic transformation of the data obtained from the 2^−ΔΔCT^ method^45^, RT-qPCR results were compared between non-osteoporotic controls, osteopaenia and osteoporotic patients using one-way ANOVA with post hoc ‘Bonferroni’s multiple comparisons test’ and two-tailed Mann Whitney test using GraphPad Prism version7.01 (GraphPad). Results were displayed as ‘Box-and-Whisker’ plots with the 25th, middle and 75th percentiles, and minimum to maximum ranges (error bars). Pearson correlation coefficient between relative expression level and lumber spine (L2-L4) *T*-score was calculated and tested. A total of 22 participants, who were under 40 years old, were not included in the data analysis, including 18 of the healthy controls, 2 of the osteopaenia and 2 of the osteoporosis patients. 12 of the non-osteoporotic participants who were over 40 years old were included in the data analysis. The diagnostic value of circulating microRNAs in serum or plasma samples was calculated using Receiver Operating Characteristics (ROC) curve analysis using SPSSS, version 22. The *P* value tests the null hypothesis that the area under the curve equals 0.5. The cut‐off points with the highest sensitivity and specificity were determined. Statistical significance was declared when the two-sided *P*-value was <0.05.

## Electronic supplementary material

Supplemental Table
