# Supplementary material for: Circulating microRNAs as potential diagnostic biomarkers for osteoporosis
Source: Sci Rep. 2018 May 30;8:8421. doi: 10.1038/s41598-018-26525-y (PMC5976644; doi:10.1038/s41598-018-26525-y)
Supplement: Supplementary file 1 — Supplemental Table [file 41598_2018_26525_MOESM1_ESM.docx]

**Circulating microRNAs as potential diagnostic biomarkers for osteoporosis**

Abdullah Y. Mandourah^1^, Lakshminarayan Ranganath^2^, Roger Barraclough^3^, Sobhan Vinjamuri^4^, Robert Van’T Hof^1^, Sandra Hamill^4^, Gabriela Czanner^5^, Ayed A. Dera^1,7^, Duolao Wang^6^, Dong L. Barraclough^1*^

**Supplemental Table S1.** **Comparisons of levels of miR-122-5p or miR-4516 in participant groups using Bonferroni ad hoc ANOVA test.** Control, osteopaenia and osteoporosis groups are compared pairwise using Bonferroni correction when all participants are included (Included all data), when patients suffering from other diseases are excluded (Excluded patients with other diseases), or males are excluded (Female only data excluding males) or patients receiving anti osteoporotic therapy are excluded (Excluded anti-osteoporotic treatments). In no case, did any of the exclusions affect the significance of the comparisons between osteopaenia or osteoporosis with the control group.

| **miRNA and group** | **Included all data** | | **Excluded patients with other diseases** | | **Female only data excluding males** | | **Excluded anti-osteoporotic treatments** | |
| --- | --- | --- | --- | --- | --- | --- | --- | --- |
| **miR-122-5p** | ***P*** | **95% CI** | ***P*** | **95% CI** | ***P*** | **95% CI** | ***P*** | **95% CI** |
| Control vs Osteopaenia | 0.158 | -0.082 to 0.501 | 0.165 | -0.085 to 0.4886 | 0.401 | -0.166 to 0.411 | 0.162 | -0.08 to 0.470 |
| Control vs Osteoporosis | 0.003 | 0.155 to 0.758 | 0.0045 | 0.141 to 0.745 | 0.009 | 0.1 to 0.697 | 0.006 | 0.128 to 0.751 |
| Osteopeania vs Osteoporosis | 0.005 | 0.077 to 0.417 | 0.0146 | 0.049 to 0.434 | 0.002 | 0.102 to 0.45 | 0.028 | 0.027 to 0.461 |
|  |  |  |  |  |  |  |  |  |
| **miR-4516** |  |  |  |  |  |  |  |  |
| Control vs Osteopaenia | 0.174 | -0.083 to 0.452 | 0.076 | -0.023 to 0.48 | 0.279 | -0.131 to 0.448 | 0.226 | -0.095 to 0.398 |
| Control vs Osteoporosis | 0.009 | 0.098 to 0.652 | 0.0005 | 0.219 to 0.755 | 0.035 | 0.023 to 0.626 | 0.008 | 0.103 to 0.657 |
| Osteopaenia vs Osteoporosis | 0.016 | 0.037 to 0.344 | 0.0029 | 0.09 to 0.427 | 0.058 | -0.0056 to 0.337 | 0.017 | 0.041 to 0.416 |

**Supplemental Table S2. Osteoporosis-related mRNAs that are targets for hsa-miR-122-5p and hsa-miR-4516.** mRNAs targeted by both hsa-miR-122-5p and hsa-miR-4516 are shown in bold. mRNAs targets of hsa-miR-122-5p and hsa-miR-4516, which encode members of the same family of proteins are shown in italics. Other targeted mRNAs are shown in plain text. Asterisks denote mRNA encoding proteins that have been associated with activities of osteoclasts/osteoblasts.

| **hsa-miR-122-5p** | | **hsa-miR-4516** | |
| --- | --- | --- | --- |
| ALPH | Alkaline phosphatase, liver/bone/kidney* | AR | Androgen receptor* |
| ANKH | Inorganic pyrophosphate transport regulator* | **BMP2K** | **BMP2 inducible kinase*** |
| **BMP2K** | **BMP2 inducible kinase*** | CD47 | CD47 |
| CA10 | Carbonic Anhydrase 10 | *CNR1* | *Cannabinoid receptor 1** |
| CD44 | CD44* | *CYP17A1* | *Cytochrome P450 family 17 subfamily A member 1* |
| *CNR2* | *Cannabinoid receptor 2** | *CYP19A1* | *Cytochrome P450 family 19 subfamily A member 1* |
| *CYP3A4* | *Cytochrome P450 family 3 subfamily A member 4* | **FSHB** | **Follicle stimulating hormone beta subunit*** |
| ESR1 | Estrogen receptor 1* | **IGF1R** | **Insulin-like growth factor 1 receptor*** |
| **FSHB** | **Follicle stimulating hormone beta subunit*** | KIT | KIT protooncogens receptor tyrosine kinase |
| GH1 | Growth hormone 1 | LTF | Lactotransferrin |
| **IGF1R** | **Insulin-like growth factor 1 receptor*** | *MAPK3* | *Mitogen activated protein kinase 3* |
| INSL3 | Insulin-like 3 | MTHFR | Methylenetetrahydrofolate reductase |
| LRP6 | Low density lipoprotein receptor related protein 6* | PLOD1 | Procollagen-lysine,2-oxoglutarate 5-dioxygenase 1 |
| *MAPK1* | *Mitogen activated protein kinase 1* | **PTHLH** | **Parathyroid hormone-like hormone** |
| **PTHLH** | **Parathyroid hormone-like hormone** | **RUNX2** | **Runt-related transcription factor 2*** |
| **RUNX2** | **Runt-related transcription factor 2*** | SLC22A11 | Solute carrier family 22 member 11 |
| **SPARC** | **Secreted protein acidic and cysteine rich*** | **SPARC** | **Secreted protein acidic and cysteine rich*** |
| TNFRSF1B | Tumour necrosis factor receptor superfamily member 1B | THBS1 | Thrombospondin 1 |
| **TSC22D3** | **TSC22 domain family member 3*** | TRAF6 | Tumour necrosis factor receptor associated factor 6 |
| **VDR** | **Vitamin D (1,25-dihydroxy vitamin D3) receptor*** | **TSC22D3** | **TSC22 domain family member 3*** |
|  |  | **VDR** | **Vitamin D (1,25-dihydroxy vitamin D3) receptor*** |

**Supplemental Table S3. A list of miRNA primers used for RT-qPCR**

| miRNA | Primer Sequence | QIAGEN Catalogue |
| --- | --- | --- |
| hsa-miR-21-3p | 5'UAGCUUAUCAGACUGAUGUUGA | MS00009079 |
| hsa-miR-1231 | 5'GUGUCUGGGCGGACAGCUGC | MS00031290 |
| hsa-miR-100-5p | [5'AACCCGUAGAUCCGAACUUGUG](http://www.mirbase.org/cgi-bin/get_seq.pl?acc=MIMAT0000098) | [MS00003388](https://www.qiagen.com/geneglobe/miprimerview.aspx?ID=MS00003388) |
| hsa-miR-122-5p | [5'UGGAGUGUGACAAUGGUGUUUG](http://www.mirbase.org/cgi-bin/get_seq.pl?acc=MIMAT0000421) | [MS00003416](https://www.qiagen.com/geneglobe/miprimerview.aspx?ID=MS00003416) |
| hsa-miR-215-5p | [5'AUGACCUAUGAAUUGACAGAC](http://www.mirbase.org/cgi-bin/get_seq.pl?acc=MIMAT0000272) | [MS00003829](https://www.qiagen.com/geneglobe/miprimerview.aspx?ID=MS00003829) |
| hsa-miR-3911 | [5'UGUGUGGAUCCUGGAGGAGGCA](http://www.mirbase.org/cgi-bin/get_seq.pl?acc=MIMAT0018185) | [MS00023527](https://www.qiagen.com/geneglobe/miprimerview.aspx?ID=MS00023527) |
| hsa-miR-1290 | [5'UGGAUUUUUGGAUCAGGGA](http://www.mirbase.org/cgi-bin/get_seq.pl?acc=MIMAT0005880) | [MS00014518](https://www.qiagen.com/geneglobe/miprimerview.aspx?ID=MS00014518) |
| hsa-miR-194-5p | 5'UGUAACAGCAACUCCAUGUGGA | MS00006727 |
| hsa-miR-145-3p | [5'GGAUUCCUGGAAAUACUGUUCU](http://www.mirbase.org/cgi-bin/get_seq.pl?acc=MIMAT0004601) | [MS00008708](https://www.qiagen.com/geneglobe/miprimerview.aspx?ID=MS00008708) |
| hsa-let-7a-3p | 5'UGAGGUAGUAGGUUGUAUAGUU | MS00031220 |
| hsa-miR-4306 | 5'UGGAGAGAAAGGCAGUA | MS00021511 |
| hsa-miR-10b-5p | 5'UACCCUGUAGAACCGAAUUUGUG | MS00031269 |
| hsa-miR-365b-3p | 5'UAAUGCCCCUAAAAAUCCUUAU | MS00031801 |
| hsa-miR-200b-3p | 5'UAAUACUGCCUGGUAAUGAUGA | MS00009016 |
| hsa-miR-99a-5p | [5'AACCCGUAGAUCCGAUCUUGUG](http://www.mirbase.org/cgi-bin/get_seq.pl?acc=MIMAT0000097) | [MS00003374](https://www.qiagen.com/geneglobe/miprimerview.aspx?ID=MS00003374) |
| hsa-miR-3923 | 5'AACUAGUAAUGUUGGAUUAGGG | [MS00023611](https://www.qiagen.com/geneglobe/miprimerview.aspx?ID=MS00023611) |
| hsa-miR-4258 | 5'CCCCGCCACCGCCUUGG | [MS00021175](https://www.qiagen.com/geneglobe/miprimerview.aspx?ID=MS00021175) |
| SNORD61 | 5’GCTATGATGAATTTGATTGCATTGATCGTCTGACATGATAATGTATTTTTGTCCTCTAAGAAGTTCTGAGCTT | MS00033705 |
| hsa-miR-196b-3p | 5'UAGGUAGUUUCCUGUUGUUGGG | MS00031570 |
| hsa-miR-485-5p | 5'AGAGGCUGGCCGUGAUGAAUUC | [MS00006972](https://www.qiagen.com/geneglobe/miprimerview.aspx?ID=MS00006972) |
| hsa-miR-1193 | 5'GGGAUGGUAGACCGGUGACGUGC | [MS00020265](https://www.qiagen.com/geneglobe/miprimerview.aspx?ID=MS00020265) |
| hsa-miR-2467-3p | 5'AGCAGAGGCAGAGAGGCUCAGG | MS00037674 |
| hsa-miR-1281 | 5'UCGCCUCCUCCUCUCCC | [MS00014455](https://www.qiagen.com/geneglobe/miprimerview.aspx?ID=MS00014455) |
| hsa-miR-4274 | 5'CACUGUAGGUGAUGGUGAGAGUGGGCA | [MS00014105](https://www.qiagen.com/geneglobe/miprimerview.aspx?ID=MS00014105) |
| hsa-miR-4516 | [5'GGGAGAAGGGUCGGGGC](http://www.mirbase.org/cgi-bin/get_seq.pl?acc=MIMAT0019053) | [MS00037555](https://www.qiagen.com/geneglobe/miprimerview.aspx?ID=MS00037555) |
| hsa-miR-4306 | [5'UGGAGAGAAAGGCAGUA](http://www.mirbase.org/cgi-bin/get_seq.pl?acc=MIMAT0016858) | [MS00021511](https://www.qiagen.com/geneglobe/miprimerview.aspx?ID=MS00021511) |
| hsa-miR-548e-3p | [5'AAAAACUGAGACUACUUUUGCA](http://www.mirbase.org/cgi-bin/get_seq.pl?acc=MIMAT0005874) | [MS00014735](https://www.qiagen.com/geneglobe/miprimerview.aspx?ID=MS00014735) |
| hsa-miR-206 | [5'UGGAAUGUAAGGAAGUGUGUGG](http://www.mirbase.org/cgi-bin/get_seq.pl?acc=MIMAT0000462) | [MS00003787](https://www.qiagen.com/geneglobe/miprimerview.aspx?ID=MS00003787) |
| hsa-miR-548d-5p | [5'AAAAGUAAUUGUGGUUUUUGCC](http://www.mirbase.org/cgi-bin/get_seq.pl?acc=MIMAT0004812) | [MS00010136](https://www.qiagen.com/geneglobe/miprimerview.aspx?ID=MS00010136) |
| hsa-miR-373-5p | [5'ACUCAAAAUGGGGGCGCUUUCC](http://www.mirbase.org/cgi-bin/get_seq.pl?acc=MIMAT0000725) | [MS00006867](https://www.qiagen.com/geneglobe/miprimerview.aspx?ID=MS00006867) |
| hsa-miR-375 | [5'UUUGUUCGUUCGGCUCGCGUGA](http://www.mirbase.org/cgi-bin/get_seq.pl?acc=MIMAT0000728) | [MS00004088](https://www.qiagen.com/geneglobe/miprimerview.aspx?ID=MS00004088) |
| hsa-miR-450a-5p | [5'UUUUGCGAUGUGUUCCUAAUAU](http://www.mirbase.org/cgi-bin/get_seq.pl?acc=MIMAT0001545) | [MS00006937](https://www.qiagen.com/geneglobe/miprimerview.aspx?ID=MS00006937) |
| hsa-miR-143-3p | [5'UGAGAUGAAGCACUGUAGCUC](http://www.mirbase.org/cgi-bin/get_seq.pl?acc=MIMAT0000435) | [MS00003514](https://www.qiagen.com/geneglobe/miprimerview.aspx?ID=MS00003514) |
| hsa-SNORD96A | 5’CCTGGTGATGACAGATGGCATTGTCAGCCAATCCCCAAGTGGGAGTGAGGACATGTCCTGCAATTCTGAAGG | [MS00033733](http://www.qiagen.com/gb/products/catalog/assay-technologies/mirna/miscript-primer-assays?catno=MS00033733) |
| hsa-RNU6-6P | 5’[GTGCTCGCTTCGGCAGCACATATACTAAAATTGGAACGATACAGAGAAGATTAGCATGGC](http://www.ncbi.nlm.nih.gov/nuccore/NR_002752) | [MS00033740](http://www.qiagen.com/gb/products/catalog/assay-technologies/mirna/miscript-primer-assays?catno=MS00033740) |
